# Determining Goals of Care During the COVID-19 Pandemic: A Virtual Course for Emergency Medicine Residents

**DOI:** 10.7759/cureus.14558

**Published:** 2021-04-19

**Authors:** Megan E Crossman, Megan Stobart-Gallagher, Mari Siegel

**Affiliations:** 1 Emergency Medicine, Einstein Medical Center Philadelphia, Philadelphia, USA; 2 Emergency Medicine, Thomas Jefferson University Hospital, Philadelphia, USA

**Keywords:** goals of care, end of life, palliative, resident didactics, virtual learning, emergency medicine training, emergency medicine resident, covid-19, critical care and hospital medicine

## Abstract

Background

Patients’ goals of care (GOC), which direct end-of-life clinical decision-making, should be established in conjunction with their primary physician when they are well. However, these discussions are often left for when critical intervention is needed in the Emergency Department, and this has been exacerbated in the new context of the current coronavirus disease 2019 pandemic. Establishing effective, formal training for Emergency Medicine (EM) residents to successfully carry out these conversations and potentially improve patient care is needed.

Methodology

A physician dual-certified in Emergency Medicine and Palliative Care developed a virtual course on best practices in determining GOC for EM residents. It occurred for one hour during resident didactic conference and all residents who attended were included. A survey was sent to all participants to assess the success of the course’s content and method of delivery.

Results

Of the 39 residents who participated, 18 (46%) completed the survey. The majority (94%) agreed the course helped close knowledge gaps and increased comfort in carrying out these discussions and 100% planned to incorporate these learning points into practice. A smaller majority (61%) thought the virtual platform was an effective method of delivery and 61% felt the breakout rooms helped with learning retention. Open-ended comments reflected learners’ desire for more of this content, suggestions to augment with simulation, as well as technical difficulties experienced.

Conclusions

This course helped EM residents identify and close knowledge gaps in determining patients’ GOC who plan to incorporate what they learned into their clinical practice. Next steps in validating the course include seeking more pointed feedback of the virtual format and assessing its effects over broader audiences after making feedback-focused adjustments to its content and delivery.

## Introduction

Goals of care (GOC) discussions for advanced care planning should ideally occur while a patient is well, long before end-of-life decisions are required, with their primary care physician [1]. Instead, these assessments often need to be rapidly completed in the Emergency Department (ED) when patients are acutely ill and require invasive resuscitative measures to sustain their lives [2]. This is due to a multitude of reasons, particularly the discomfort associated with these conversations and the high proportion of patients who lack access to quality preventive care in the United States insurance-based healthcare system [3]. Success in facilitating patient-centered care that aligns with their goals leads to an improved experience for patients and their families [4]. These discussions can exacerbate anger and loss of trust if not performed correctly [5]. While this has been the unfortunate reality for many critical patients seen in the ED, the issue has been acutely exacerbated by the emergence of the novel coronavirus disease 2019 (COVID-19) pandemic by increasing the total pool of patients with critical illness and preventing family members from being present for these conversations in person [6].

The purpose of a physician initiating a GOC discussion is to assess the patient’s values and beliefs. This information is then used to formulate end-of-life decisions congruent with these goals. Rather than initiating the conversation by simply asking if patients would want intubation or cardiopulmonary resuscitation (CPR), it is paramount to discover what is most important to them so that the physician may frame their options in this context [6]. This course provides learners with resources including scripts to facilitate their carrying out this task as well as provide them with the opportunity to practice utilizing these tools. The content was delivered in a virtual video conference format to adhere to social distancing rules secondary to the COVID-19 pandemic.

Although emergency physicians are expected to undergo this difficult task under high-stakes circumstances, it is inconsistently taught during mandatory didactics or simulated, and thus can be considered to be part of a residency’s “hidden curriculum” [7]. Residents would benefit from formal training in how to successfully carry out these discussions as part of their regular didactics conference [8]. When the high morbidity and mortality associated with intubation in the setting of COVID-19 infection became apparent, it further emphasized the need to implement formal training for our residents in engaging in GOC discussions [9]. In response to this emerging need, a course was designed specifically for Emergency Medicine (EM) residents by a dual-boarded faculty member in both Emergency Medicine and Palliative Care to fill this educational void.

The primary objective of this session was to provide formal training to EM residents in the initiation of GOC discussions while providing critical care to acutely decompensating patients during the COVID-19 pandemic.

## Materials and methods

This activity lasted approximately one hour and took place during weekly EM residency didactics conference at an urban tertiary hospital in the Northeast United States. The content was delivered via Zoom video conferencing application (https://zoom.us/) to correspond with our conferences and meetings, which had been shifted to the virtual realm due to the COVID-19 pandemic’s social distancing guidelines. The course began with an informative slide-based talk to provide resources and educational content that would then be practiced in small groups using Zoom’s breakout room function, which separates learners into smaller groups for deeper discussion. The presentation introduced the concepts of informed assent as opposed to informed consent. Informed assent posits that when a physician believes that CPR or intubation is extremely unlikely to benefit the patient and will not achieve the patient’s known goal of quality of life or comfort, it is appropriate to not offer CPR or intubation as an option [6]. The core content also covered two different frameworks for patient and physician goal setting.

The session began by outlining the two different frameworks for goal setting. The first introduced was GUIDE, which is a tool developed by Vital Talk to help remember five key steps in carrying out these difficult discussions (Table 1). The GUIDE process is appropriate for patients whose advanced care planning and code status have already been determined prior to the encounter [10]. It starts with “getting ready” by securing a quiet space, minimizing interruptions, and gathering important friends and/or family. The next step is to understand the patient and what they know about their illness. After this, the physician is to inform and align the patient as to their current medical situation. They should then demonstrate empathy, which can be assisted by the acronym NURSE as this can be particularly difficult to convey appropriately in situations of low likelihood of survival [10,11]. Lastly, the provider should equip the patient with the relevant information to make informed GOC decisions and end the conversation by explaining next steps (Appendix 1).

**Table 1:** GUIDE, a tool for breaking bad news [10]. GUIDE: Get ready, Understand, Inform, Demonstrate empathy, Equip

Step	What to say or do
Get Ready – Info, People, Place	Make sure you have all the information. Make sure you have all the right people present. Find a place with privacy.
Understand what the patient knows	What have you taken away from other doctors so far?
Inform starting with a headline	Give the information clearly and to the point with a one-sentence headline. Avoid jargon. After the headline you will need to give more information, but after giving the headline, STOP!
Demonstrate empathy and respond directly to emotion	“I can see this news is not what you were hoping for.” Expect the patient’s first response to be emotion. Acknowledge the emotion explicitly.
Equip the patient for the next step	“I want you to be prepared for the next step. Can I explain…” Don’t dismiss concerns or say that everything will be fine.

The second outline to facilitate the GOC discussion is REMAP, which is appropriate in situations when the patient’s end-of-life preferences are undetermined (Table 2). Similar to GUIDE, this is an acronym with each letter standing for five key steps. It begins with reframing and reorienting the patient with the new diagnostic and prognostic information. Next, the clinician should expect emotion and give the patient space for them to express this. After providing the patient a moment to absorb and respond to the new information, the physician should then work towards mapping out the patient’s goals. The three main options include longevity, quality, and comfort. The following step is to summarize and assure this aligns with the patient’s values. The process closes with deciding on a plan that is congruent with what was discussed [12].

**Table 2:** REMAP, an outline to facilitate GOC discussions [12]. REMAP: Reframe, Expect emotion, Map, Align, Plan; GOC: goals of care

Step	What to say or do
Reframe why the status quo isn’t working	You may need to discuss serious new results first. “Given this news, it seems like a good time to talk about what to do next.”
Expect emotion and empathize	“It’s hard to deal with all this.” “I can see you are really concerned about [x].” “Tell me more about that—what are you worried about?”
Map the future	“Given this situation, what is most important for you?” “When you think about the future, are there things you want to do?” “As you think towards the future, what concerns you?”
Align with the patient’s values	“As I listen to you, it sounds the most important things are [x,y,z].”
Plan medical treatments that match patient’s values	“Here’s what I can do now that will help you do those important things. What do you think about it?”

Resident physicians were then divided into small groups of five or six where they had the opportunity to practice using the GUIDE and REMAP frameworks with predetermined case presentations. Residents were given scripts and the role played, either the physician or family members, in two cases where GOC need to be established in the acute setting. Each group was facilitated by faculty members who received a brief pre-session training in the above frameworks to guide reflection after the cases. After this 20-minute role play session, the groups reconvened as a whole to debrief. The remainder of the time focused on the COVID-19 pandemic specifically, as well as on how GOC discussions may occur virtually and in less than ideal circumstances.

Following the course, an anonymous survey was sent to all participants utilizing Survey Monkey (https://www.surveymonkey.com/), the responses of which were then tabulated and analyzed after giving learners approximately two months to complete it, as we were not receiving any additional responses at this point.

## Results

A total of 39 out of 51 EM residents attended this session. A survey that included five closed-ended questions using the Likert scale with five options from strongly disagree to strongly agree and one open-ended question was sent out to all the participants. We received 18 (46%) responses, all of which completed the entire survey. Overall, the responses were positive. The first three closed-ended questions asked questions related to content and whether it improved their knowledge relating to this topic. Overall, 17 (94%) either agreed or strongly agreed that they feel more comfortable discussing GOC and that the course helped them identify and close knowledge gaps regarding this topic. All participants (100%) either agreed or strongly agreed that they plan on incorporating what they learned into their clinical practice (Table 3).

**Table 3:** Close-ended survey responses assessing course content. GOC: goals of care; COVID-19: coronavirus disease 2019

Survey prompt	Strongly agree	Agree	Neither agree nor disagree	Disagree	Strongly disagree
After completing this course, I feel more comfortable discussing GOC with COVID-19 patients and their loved ones	3 (16%)	14 (78%)	1 (6%)	0 (0%)	0 (0%)
This course helped me discover and close any knowledge gaps in GOC discussions	6 (33%)	11 (61%)	1 (6%)	0 (0%)	0 (0%)
I plan on incorporating what I learned from this course into my clinical practice	5 (28%)	13 (72%)	0 (0%)	0 (0%)	0 (0%)

The remaining two closed-ended survey questions, which assess the course’s format, also received overall positive responses. However, the responses were more mixed. Overall, 11 (61%) either agreed or strongly agreed that video conference was an effective mode of delivery for this course. The remaining seven stated they were neutral. In total, 12 (67%) respondents were in agreement that the small group activities helped them retain the information from the lecture portion, whereas five (28%) were neutral and one (5%) disagreed (Table 4).

**Table 4:** Close-ended survey responses assessing course format.

Survey prompt	Strongly agree	Agree	Neither agree nor disagree	Disagree	Strongly disagree
Zoom video conference was an effective method of delivery for this course	1 (6%)	10 (55%)	7 (39%)	0	0
Small group activities (breakout rooms) helped me retain the content of this course	2 (11%)	10 (55%)	5 (28%)	1 (6%)	0

The single open-ended question asked participants to give one additional comment, feedback, or suggestion for improvement. The majority were positive reviews of the course as well as requests for more sessions like this one. Some commented that the breakout rooms were “clunky” and “did not work well on zoom,” but others stated the small groups were “helpful” and “really helped with retention” despite technical difficulties. Unfortunately, there were no further specifics regarding technical issues. There were also some suggestions of items and activities that could add to the course in a beneficial way such as more “hands-on SIM sessions,” handouts, as well as including additional frameworks that facilitate GOC discussions (Table 5).

**Table 5:** Responses to open-ended prompt requesting feedback.

Survey prompt	Theme	Examples of responses
Please write at least one additional comment, feedback or suggestion for improvement	Small group session	I hope that we will schedule more of these sessions, and I would like to have a practice session for residents in addition to the small group practice sessions that were already done for the attendings
		The small groups where we could simulate discussions were helpful in allowing us to practice in a small group (less intimidating)
		The small group breakout rooms were essential to the format and really helped with retention. Some technical difficulties made it clunky at times but did not detract significantly from the learning
		Breakout sessions great idea but did not work well on ZOOM
	Supplemental modalities of learning	More hands-on sim sessions
		Handout after with simple overview
	Why this was helpful	It was nice learning about the 2 different methods, one for an initial GOC and another for a quick updated GOC
		Very helpful session overall. I think it is really important to give us key phrases to use in these stressful and highly charged emotional situations. If I practice using these specific phrases, I will be more comfortable falling back on them for clinical use
		More of this content!

## Discussion

Although rapidly assessing critically ill patients’ GOC is a difficult skill that requires practice, formal training is not always included in traditional didactics. This void has been emphasized by the increased number of critically ill patients due to the COVID-19 pandemic. Our results show that this course was an overall success in improving knowledge, skill development, and its educational format chosen for distribution.

Of the five closed-ended questions, the majority received positive responses. This was most pronounced in those relating to medical content, none of which had a negative response of disagree or strongly disagree. All respondents reported that they planned to incorporate the framework approaches learned into their current practice. While the questions related to course structure received more neutral responses, the majority were positive and only one response was negative or “disagree.” The open-ended statements reflected the general sentiment of the results from the close-ended questions, mostly expressing that the course was helpful and beneficial, with some commenting on some drawbacks to the video conferencing format. They also included some great suggestions as to how the course may be improved, including more opportunities to practice in a simulated format.

This study carries a few limitations. Although every effort was made to have all learners who attended complete a survey, only 46% did. The fact that less than 50% of those who participated evaluated the course means we cannot conclude the overall positive response reflected the majority. Additionally, the data are subjective and lack any objective, measurable findings as to whether the course successfully imparted the knowledge presented to learners. This was also a single-center study, which may limit generalizability to other institutions or geographic areas.

Next steps in order to address these limitations are to repeat the course for other learners to expand the data pool and improve the completion rate of evaluations. Future studies should include pre- and post-intervention assessments, the improvement of which would objectively validate the efficacy of this course. New adaptations of the course that utilize different learning methods other than role play should also be evaluated to see if there are more successful alternative models. A simulation session could be an appropriate arena for this, which was one of the suggestions offered by our respondents. Our institution does hold an annual “bad news SIM” for all residents; however, this was interrupted by social distancing requirements. Future surveys should also request more specifics about any technical issues. Although this was mentioned, none of the comments stated any details regarding solutions to this problem. No issues were made apparent in real time to the presenter, but one possibility is that the small groups felt unorganized or without clear direction. This could be addressed by distributing more detailed outlines to facilitators in advance as well as more thoroughly orienting learners to the activity’s objectives in the large group just prior to starting this portion.

## Conclusions

Our results evaluating this novel course indicate that it successfully helps learners identify and close their knowledge gaps in leading GOC discussions and improve their comfort level in broaching this intimidating topic with patients and their families. This can be successfully incorporated into a virtual residency didactic curriculum.

## Appendices

Case utilizing GUIDE

Mrs. Smith is an 85-year-old female with a history of heart failure with an ejection fraction of 30% s/p AICD, CKD stage 2, and mild COPD who lives at home with her husband. She is independent with her activities of daily living. She presents to your Emergency Department complaining of cough and fever. Her oxygen level on room air is 78% and she is placed on a non-rebreather mask but does not significantly improve. Her exam reveals crackles at the right base and chest X-ray shows a lobar pneumonia. You call for BiPAP and sit down with Mrs. Smith to discuss her condition and explore her goals.

GET READY

[Key Information] [Key People] [Key Space]

UNDERSTAND what they know

[Warning Statement]

Physician: “I have some serious news to talk about today.”

[Assess Prior Knowledge]

Physician: “So, to know where to begin, it’s helpful to know what you’ve already been told. What do you already know about your condition today?

Patient: Well doctor, I know I have a bad heart and today I am having trouble breathing.

[Ask Permission]

Physician: May I tell you what I know?

INFORM using a headline

[Headline = Information + Meaning]

Information (1-2 sentences of key information):

Physician: Your X-ray and CT scan results show that you have an infection in your lungs. The infection looks pretty bad, and I am worried that you won’t be able to breathe on your own much longer. I need to talk to you about ways I can help you breathe, either by having you wear a face mask or by having you go to sleep, so we can place a tube into your lungs.

Stop! Respond to emotions before giving more medical information.

DEMONSTRATE EMPATHY

Physician: This must be hard news to hear. I can only imagine how difficult this is to think about.

Patient: Oh wow. This is so scary. Thank you for telling me. Listen, a few years ago I had a bad heart failure exacerbation and I was on a ventilator for a long time. It took me a long time to recover. I don’t want to go through that again.

EQUIP for next steps

[Align First]

Physician: I want you to know that our team will do everything we can to support you.

[Anticipatory Guidance]

Physician: I also want you to be prepared for what’s to come. Our plan right now is to help you breathe with a machine called BiPAP. It is a mask that goes over your face, but you are awake. We will admit you to the hospital. Sometimes people’s condition worsens, despite our best efforts. Given what you just told me, if you become critically ill we will not put a tube into your lungs and connect you to a ventilator machine that breathes for you. Instead we would shift our focus to keeping you comfortable. Does that sound right to you?

Patient: Yes doc, that’s right. I want to live, and I hope I get better, but I don’t want to be on a ventilator. Thank you.

[Check-in]

Physician: That is a lot to process, what questions do you have?

Patient: Can my family come and visit me now?

[Affirm and Close]

Physician: Thank you for talking with me about this today. I will write our discussion down in your chart, so everyone on our healthcare teams knows the plan. We are committed to making sure you get the best care possible.
